# Implementing primary health care-based PMTCT interventions: operational perspectives from Muhima cohort analysis (Rwanda)

**DOI:** 10.11604/pamj.2014.18.59.3895

**Published:** 2014-05-17

**Authors:** Maurice Bucagu, John Muganda

**Affiliations:** 1 World Health Organization Department of Maternal, Newborn, Child and Adolescent Health, Geneva, Switzerland; 2 King Faisal Hospital, Department of Obstetrics & Gynecology, Kigali, Rwanda

**Keywords:** PMTCT implementation, primary health care, operational considerations, integrated service delivery, task shifting, community-based health insurance, cohort, Rwanda

## Abstract

**Introduction:**

In countries with high burden of HIV, major programmatic challenges have been identified to preventing new infections among children and scaling up of treatment for pregnant mothers. We initiated this study to examine operational approaches that were used to enhance implementation of PMTCT interventions in Muhima health Centre (Kigali/Rwanda) from 2007 to 2010.

**Methods:**

The prospective cohort study was conducted at Muhima health centre. A sample size of 656 was the minimum number required for the study. The main outcome was cumulative incidence of mother - to - child transmission of HIV-1 measured at 6 weeks of life among live born children.

**Results:**

Among the 679 live born babies and followed up in this study, the overall cumulative rate of HIV-1 mother - to - child transmission observed was 3.2% at 6 weeks of age after birth. Disclosure of HIV status to partner was significantly associated with HIV-1 status of infants at 6 weeks of age (non-disclosure of HIV status adjusted odds ratio [AOR] 4.68, CI 1.39 to 15.77, p.

**Conclusion:**

The Muhima type of decentralized health facility offered an appropriate platform for implementation of PMTCT interventions, with the following operational features: family - centered approach; integrated service delivery for PMTCT/MCH interventions, task shifting; subsidized membership fees for people living with HIV, allowing for access to the community-based health insurance benefits.

## Introduction

After thirty years of AIDS pandemic, the world has achieved significant progress towards preventing mother-to-child transmission (MTCT) of HIV. The 2013 UNAIDS global report shows a 52% decline in new HIV infections among children between 2001 and 2012 [1]. Despite these global accomplishments, the mother - to - child transmission of HIV remains a major challenge in Sub-Saharan Africa (SSA) where more than 90% of children who acquired HIV infection live. It is estimated that “pregnant women living with HIV are less likely than treatment-eligible adults to receive antiretroviral therapy” [2].

The recent HIV developments provided a critical window of opportunity for the global community to commit to the goal of eliminating new HIV infections among children and keeping their mothers alive, with the following international benchmarks: overall transmission rate [3].

In this context, the 2013 WHO guidelines on the use of antiretroviral drugs for treating and preventing HIV infection, provide great opportunities for simpler and safer ARV regimens, based on new evidence, for use in most populations. “ART is recommended to be initiated as prevention regardless of CD4 count for pregnant and breastfeeding mothers”. In addition, relevant guidance is provided to programme managers to ensure the long-term effectiveness and sustainability of ARV programmes, with special focus on the following key operational aspects: adherence to ART; retention across the continuum of care; service delivery, comprising service integration and linkage and decentralization of HIV, care and treatment; human resources including task shifting; laboratory services; procurement and supply management systems [4].

In countries with high burden of HIV, major programmatic challenges have been identified to preventing new infections among children and scaling up of treatment for pregnant mothers. And it is anticipated by UNAIDS that Integrating comprehensive prevention and antiretroviral services with maternal, neonatal and child health services, in the context of continuum of care, would minimize the number of pregnant women who drop out of services. This would improve the efficiency and effectiveness of PMTCT related interventions, particularly in countries with generalized HIV epidemics [1].

Interventions for prevention of mother-to-child transmission of HIV-1 (PMTCT) in Rwanda were first piloted as stand-alone services in 1999. Since 2004, the country moved from a “ vertical PMTCT model” to an “integrated” approach with the goal of building a more cost-effective and sustainable system. And there were reports on significant progress towards scaling up of PMTCT key interventions, including the use of antiretroviral medicines, through decentralized service delivery, with an increasing uptake of HIV testing and counselling from 27.1% in 2004 to 73% in 2009, among pregnant women attending ANC services [3].

From the Rwanda Demographic and Health Survey (DHS, 2010), it was estimated that among women, knowledge about HIV transmission from mother to child during delivery and through breastfeeding, increased respectively from 85% (2005) to 96% (2010) and from 80% (2005) to 94% (2010). The HIV prevalence among adults aged 15 to 49 was estimated at 3% (3.7% among women and 2.2% among men) [5].

The PMTCT interventions in Rwanda were implemented at primary health care level and integrated into MCH services at a critical time when these were overburden, with the rapid increase in the utilization of antenatal care services (4 visits + ) by five times and birth in health facilities from 28% to 68% reported at national level between 2005 and 2010 [6]. During the study period, ART and prophylaxis regimen were used, based on zidovudine, lamivudine and nevirapine. The Muhima health centre had to address major challenges not only in terms of the ability to reaching out to the population in need in an urban area, but also to ensure adequate adherence to ARVs and quality of care. Hence the need to enhance the MCH platform for it to be able to accommodate the PMTCT - related package of interventions with specific operational implications.

We initiated this study to examine operational approaches that were used to enhance implementation of PMTCT interventions in Muhima health Centre (Kigali/Rwanda) from 2007 to 2010. The study site is a public health facility located in the urban area of Nyarugenge district (287,529 inhabitants, 2010) [7] and was among the first public health centres considered for hosting integrated comprehensive PMTCT interventions into MCH services [3, 8, 9].

## Methods

### Study design and population

The prospective cohort study was conducted at Muhima health centre (Kigali/Rwanda). All pregnant women diagnosed with HIV-1 and attending PMTCT service at Muhima health centre were invited to participate in the study, between May 2007 and April 2010. Eligible participants were pregnant HIV-1 infected women, consenting to the study, who had attended antenatal visits or delivered at Muhima maternity and had benefited from PMTCT interventions in line with the national guidelines. Additional inclusion criteria was for participants to be registered as residents within the specific catchment area of Muhima health centre and expected to attend postnatal follow up as required. All HIV negative pregnant women, those whose consent was not obtained and those living outside Muhima catchment area were excluded from the study.

We estimated the sample size based on anticipated HIV-1 infection of 4% at 6 weeks and absolute precision in% points of 1.5, with a confidence interval (CI) of 95%. A sample size of 656 was the minimum number required for the study. During the study period, of 8,669 pregnant women who attended antenatal visits and screened for HIV-1 in Muhima health centre, 736 were found to be infected with HIV-1 and among them 700 were eligible study participants. At enrolment, these were interviewed by three trained PMTCT providers (2 data collectors supervised by 1 medical doctor) until the determined sample size of 700 women was reached. Information was collected from each mother - infant pair, including specific socioeconomic characteristics, clinical and biological features. For twins, one member randomly selected from each twin pair was included in the study.

### Data collection and management

Follow up data for eligible mother-infant pairs, about pregnancy, childbirth and postnatal period were obtained from women themselves and log books in Muhima health centre, using a structured questionnaire, translated into Kinyarwanda by the principal investigator. Those data included medical records and laboratory tests results. Required data were collected anonymously, using participant's unique identifier, nationally provided by the National Centre for Treatment and Research on AIDS, Malaria, Tuberculosis and other epidemics (Rwanda TRAC plus/Rwanda Ministry of Health).

The study was designed to allow for periodic re-questioning of study participants, at birth and 6 weeks after childbirth [10]. Study participants were considered lost to follow up if they have not shown up for regular visits and the study team unable to find HIV-1 test results for their infants at 6 weeks (Dried Blood Spot method using PCR technique) [11]. Baseline data on known variables, namely age, marital status, maternal education, residence, wealth index were found to be sufficiently similar to those of participants (679) who remained in the study. The data were double entered for all 700 questionnaires, by a team of 2 data entry clerks supervised by a lecturer from the University of Rwanda / School of Public Health. The quality control was meant for checking data consistency between the study questionnaires and medical records from the health facility where childbirth took place. The main outcome was cumulative incidence of mother - to - child transmission of HIV-1 measured at 6 weeks of life among live born children [11].

### Data analysis

Background data were summarized with descriptive statistics of the following socioeconomic characteristics: mother's age in years (< =24 years / > 24 years); marital status (married/unmarried); mother's education (no education/primary education; secondary school and more); wealth index re-categorized in 5 quintiles (poorest/second/middle/fourth/richest) (Demographic Health Survey wealth index model) [12]; parity (primiparous/multiparous). Univariate analysis of associations was performed using the chi squared test, Fisher's test as appropriate. For this, key PMTCT-related indicators were considered as covariates, including: disclosed HIV status to partner (no/yes); type of ARV treatment (prophylactic/curative); duration of ARV treatment prior to delivery (< 6 weeks / > = 6 weeks); place of delivery (home/facility); mode of delivery (vaginal/instrument assisted/cesarean section); CD4 count (< 350 cells/µL > = 350 cells/µL); child sex (male/female) and infant feeding choices (mixed feeding /artificial feeding /exclusive breastfeeding) [11]. A predictive modelling was applied with predictors of mother-to-child transmission of HIV-1 assessed by multivariable logistic regression. All variables with potential association with the main outcome - HIV-1 transmission, were entered into the logistic regression model. Hosmer and Lemeshow test was applied to check for how well the model fit. Variables were held in the model if they reached a significance level of P

**Figure 1:**
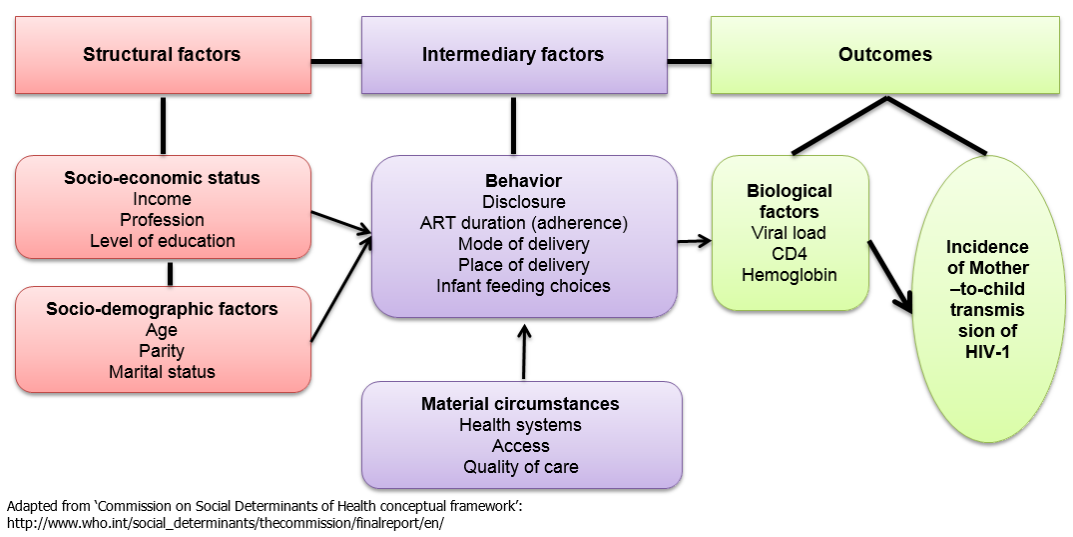
Conceptual framework of socio-economic, clinical and biological risk factors for mother – to – child transmission of HIV-1

Based on the national key policies that were implemented in the country, with effects on implementation of PMTCT-related interventions [13], specific Operational considerations (service delivery, service providers, financial access; laboratory tests, data management) were discussed, in relation to the analysed PMTCT indicators (Figure 1).

### Ethical considerations

The study protocol was reviewed and approved by the Rwanda national ethical committee and the research commission of University Teaching Hospital of Kigali, in February 2007, with annual evaluation of the study progress. An informed consent has been obtained, with a written and signed document for each study participant.

## Results

### Background characteristics

Table 1 presents the socioeconomic characteristics of mother-infant pairs enrolled in the Muhima cohort study. Nearly 1 in 3 mothers (29.7%) were young, with a median age of 27 years (range: 17 - 45 years). Majority of mothers had only primary or no education (75%) and were married (82.7%). Over two participants in five (40.3%) were classified by wealth index quintiles as poorest and poor; over one in five (21.5%) mothers were primiparas, with median number of 2 births (range: 1-9 births).

**Table 1:** Background characteristics of mothers meeting inclusion criteria in the Muhima cohort study [13]

Variables	Number (N = 679)	Percentage
**Mother's age in years**		
< = 24	202	29.7
> 24	477	70.3
**Marital status**		
Unmarried	117	17.3
Married	562	82.7
**Mother's education**		
None/primary	509	75.0
Secondary/university	170	25.0
**Wealth**		
Poorest	175	25.8
Poor	99	14.5
Middle	143	21.1
Rich	145	21.4
Richest	117	17.2
**Parity**		
Primipara	146	21.5
Multiparous	533	78.5

### PMTCT services use - related indicators [13]

**Figure 2:**
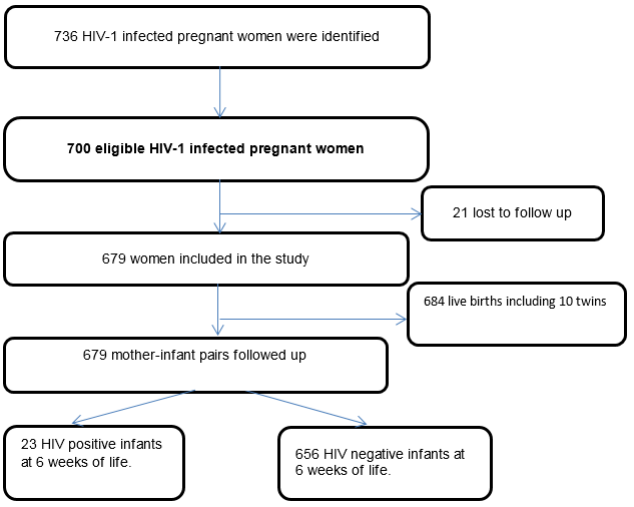
Muhima cohort profile, 2007 – 2010 (Rwanda)

Among the 679 live born babies and followed up in this study, the overall cumulative rate of HIV-1 mother - to - child transmission observed was 3.2% at 6 weeks of age after birth. Of the 700 HIV-1 infected mothers and their newborn enrolled in the study, 21 were lost to follow up (3%) (Figure 2).

Table 2 shows key PMTCT service use- related indicators. For 81.1% of study participants, male partner's HIV status was known and disclosed. Less than half of the mothers (48.2%) reported taking ART, with majority (51.8%) using ARV prophylaxis during pregnancy. For over one in three participants (22.5%), the duration of ART/ARV prophylaxis prior to childbirth was less than 6 weeks. The vast majority of babies (97.1%) were born in facility, with 82.2% of vaginal and or instrument - assisted delivery and 17.8% caesarean section rate. At enrollment, 38.7% of participants were below 350 cells/mm^3^ CD4 count level; with median CD4 count of 429.50 cells/mm^3^ (range: 11 - 1718 cells/mm^3^). The vast majority (86.1%) of mothers reported exclusive breastfeeding for their children; replacement and mixed feeding accounting respectively for 11.8% and 2.1%. Among the babies born to HIV-infected in Muhima, 50.7% were females.

**Table 2:** Univariate analysis of PMTCT service use–related coverage indicators by infant HIV-1 status at 6 weeks of life among participants in Muhima cohort study [13]

Maternal indicators	Infant HIV-1 status at 6 weeks of life			
	n/%	HIV–(%)	HIV+ (%)	P-value
**Disclosed HIV status to partner**				
No	128(18.9)	89.8	10.2	0.000
Yes	551(81.1)	98.2	1.8	
**Type of ARV treatment**				
Prophylactic	352(51.8)	97.4	2.6	0.215
Curative	327(48.2)	95.7	4.3	
**Duration of ARV treatment prior to childbirth**				
Less than 6 weeks	153(22.5)	93.5	6.5	0.014
6 weeks or more	526(77.5)	97.5	2.5	
**Place of delivery**				
Home	20(2.9)	90.0	10.0	0.097
Facility	659(97.1)	96.8	3.2	
**Mode of delivery**				
Vaginal/instrument assisted	558(82.2)	96.4	3.6	0.542
Cesarean section	121(17.8)	97.5	2.5	
**CD4 count**				
<350 cells/mm^3^	263(38.7)	94.3	5.7	0.009
≥350 cells/mm^3^	416(61.3)	98.1	1.9	
**Newborn/ infant indicators Child sex**				
**Child sex**				
Male	335(49.3)	96.7	3.3	0.883
Female	344(50.7)	96.5	3.5	
**Type of infant feeding**				
Mixed feeding	14(2.1)	85.7	14.3	0.070
Exclusive breastfeeding	585(86.1)	96.8	3.2	
Replacement feeding	80(11.8)	97.5	2.5	

In univariate analysis, among the 679 HIV exposed live infants at 6 weeks, there is a statistically significant association between HIV-1 status of infants and the following variables related to the use patterns of PMTCT services: disclosure of HIV status to partner; duration of ART/ARV prophylaxis prior to childbirth and CD4 count (Table 2)

Among the factors considered for the multivariate analysis (Table 3), only disclosure of HIV status to partner was significantly associated with HIV-1 status of infants at 6 weeks of age (non-disclosure of HIV status adjusted odds ratio (AOR) 4.68, CI 1.39 to 15.77, p < 0.05; compared to disclosure). The CD4 count level <350 cells/mm^3^ was associated with higher risk of MTCT at 6 weeks, however the statistical significance was borderline (<350 cells/mm^3^, AOR 4.83, CI 0.98 to 23.90, p < 0.1, compared to > = 350 cells/mm^3^).

**Table 3:** Multivariate analysis of PMTCT service use–related indicators of MTCT of HIV-1 at 6 weeks of life [13]

	Infant HIV-1 status at 6 weeks of life
Variables	Adjusted OR [95% CI]
**Disclosed HIV status to partner**	
No	4.68** [1.39 – 15.77]
Yes	1
**Type of ARV treatment**	
Prophylactic	1.82 [0.38 – 8.73]
Curative	1
**Duration of ARV treatment prior to childbirth**	
Less than 6 weeks	2.24 [0.81 – 6.16]
6 weeks or more	1
**Place of delivery**	
Home	2.98 [0.42 – 21.17]
Facility	1
**Mode of delivery**	
Vaginal/instrument assisted	0.66 [0.17 – 2.57]
Cesarean section	1
**CD4 count**	
<350 cells/mm^3^	4.83* [0.98 – 23.90]
≥350 cells/mm^3^	1
**Child sex**	
Male	1.27 [0.49 – 3.34]
Female	1
**Type of infant feeding**	
Mixed feeding	5.5 [0.40 – 74.82]
Exclusive breastfeeding	2.45 [0.39 – 15.24]
Replacement feeding	1

** p < 0.05

* p < 0.1.

The following variables were considered for inclusion in the logistic regression model, using stepwise variable selection: mother's age; marital status; wealth index; parity; HIV status disclosure to partner; type of ARV treatment received; duration of ARV prophylaxis/treatment; place of delivery; mode of childbirth; CD4 count; child's sex; infant feeding choices

The following national policies were implemented in Rwanda with specific effects on implementation of HIV & PMTCT - related interventions at primary health care level [14]: Decentralization of PMTCT service package, including laboratory tests (2004); Integration of PMTCT interventions into existing MCH services (2004); Family-centred approach (including strategies for effective couple counselling (2004); Community health programme enhanced (2008); Task shifting (2009); Community - based health insurance (with specific provision for support to the people living with HIV) (2005); Data management using information technology platform (TRACnet 2005).

## Discussion

Implementing PMTCT key interventions in Muhima health centre required supportive operational features as part of key functioning health systems building blocks for maximizing health benefits for mothers, children and their families [14, 15].

**Effect of PMTCT interventions:** The observed HIV-1 MTCT rate at 0-6 weeks (in utero or during intrapartum period), among the 679 babies born to HIV infected mothers in Muhima health centre, was found to be far lower (87.5% reduction in HIV transmission) than with no intervention, before the era of HAART, with HIV-1 MTCT that had affected more than one in four children born to HIV-infected mothers in Rwanda (25.7%: 95% CI 18.8 - 32.5) [16]. The Muhima study findings were similar to those published in 2012 by the national PMTCT programme, with an estimated HIV prevalence of 4% (95% CI: 2.9 - 5.0), among children aged nine to 24 months [17, 18]. The HIV-1 MTCT rate from the Muhima study was also comparable to rates reported in several studies, with similar regimen in sub-Saharan African countries. The Kesho Bora multi - centre study (Burkina Faso, Kenya and South Africa) reported a cumulative transmission rate of 3.3% (95% CI 1.9 - 5.6%) in the triple antiretroviral group, compared with 5.0% (3.3 - 7.7%) in the zidovudine and single-dose nevirapine group at 0-6 weeks [19]. In a retrospective cohort study conducted in south - south region of Nigeria, the transmission rates for mother - baby pairs who received ARVs for PMTCT were 4.8% ( CI 1.8, 8.3) at zero to 6 weeks of age compared to 19.5% (CI 3.0,35.5) in a group with no intervention for prevention [20]. Torpey et al. in Zambia observed HIV MTCT transmission rates, between zero and 6 weeks, of 6.8% (4.5%,9.1%) when Zidovudine + Nevirapine as compared to 5% (3.0%, 7.0%) with highly active antiretroviral therapy and 8.5% (5.9%,11.0%) with single dose Nevirapine. These estimates were not significantly different from one another; however they were all significantly lower than with no intervention (20.9%) [21]. In South Africa, Mnyani et al, cited by Anoje et al, reported an overall transmission rate of 5.8% for HIV exposed babies between four and six weeks of age, where mother and baby received some form of ARVs for PMTCT [19].

**Integrated service delivery:** The Family - centered approach was applied for delivering PMTCT interventions to the HIV - infected mothers, their children & families, in Muhima health centre during the study period (2007-2010) [22]. The essential PMTCT interventions were decentralized and integrated into Maternal and Child Health services, in the context of continuum of care [23, 24]. The package of services included antenatal visits, essential childbirth and postnatal care for the mother and newborn, support to adequate newborn/infant feeding practices and family planning choices as per national recommendations. Antenatal visits were used as key entry point for PMTCT implementation, with all pregnant women offered provider-initiated HIV testing & counseling and encouraged to come with their partners. These would also receive personal invitation letters issued by the health center for couples HIV testing & counselling, with same - day results & disclosure of the HIV status as standard practice. ARV drugs were prescribed for infected pregnant women and later for newborn according to the national guidelines.

In this context, a high uptake of HIV provider - initiated testing & counselling has been registered as widely documented in Rwanda [25] At national level, the uptake of HIV testing & counselling for male partners through ANC service increased from 32.5% in 2005 to 84.3% in 2011[22] In addition, utilization rate for institutional childbirth among study participants (98%) was higher than the national average (68.9%) even in urban areas (82%) [6]. Women with obstetric and/or HIV/AIDS complications were referred to the Muhima district hospital for relevant management. Caesarean section was performed in 17.8% of all deliveries among study participants. This utilization rate was comparable to that of the richer urban section of the population (17.53%) [26, 27]. High risk mixed feeding practices for HIV-1 exposed infants [28] were self-reported in only 2.1% of study participants, as opposed to exclusive breastfeeding as recommended for babies born to HIV-1 infected mothers for the first six months of life and the most common infant feeding practice in Rwanda [6]. To support lactating mothers and families with low social economic status, nutritional advice and food supplements were provided on a monthly basis (and for children after 6 months of age). Although integration of PMTCT interventions into MCH platform has been widely adopted as an appropriate approach to bring key services closer to the users, the 2013 UNAIDS report on the global epidemic stated that forty-three countries out of 118 (40%) reported having linkages or integrated service delivery between services to prevent mother-to-child transmission and broader maternal and child health services [2]. For Suthar AS et al., who reviewed the evidence surrounding the effect of integrating ART with ANC and MCH clinics on programmatic and patient outcomes, the available data suggest that integrating ART into ANC clinics is feasible and improves ART coverage [29].

**MCH/PMTCT service providers:** The Muhima - based PMTCT services were all provided by existing MCH staff, including midwifes, nurses & social workers as per national standards, in line with the national policy on task shifting. This was approved in 2009 to provide the legal framework for initiation of HAART by nurses at the decentralized level and operationalized through specific in-service training and mentoring guide to ensure that required competences and skills are acquired [29–31].

In addition, Muhima - based community health workers, with specific maternal and child health focus, each one covering about 100 households and provided with mobile phones, played a critical role in psycho-social and adherence to ART prophylaxis/treatment support for women living with HIV, their partners & children. The rapidly increasing mobile phone penetration rate of 70% & 40% respectively in urban and rural areas in Rwanda (2010) [6] provided a great opportunity for community health workers to specifically support communication, referral system, linkages between individuals, families & communities and relevant PMTCT service providers [32].

Several studies have shown that fundamental health-systems issues such as staffing and service accessibility, along with community - level factors of stigma, fear of disclosure and lack of partner support continue to emerge as major barriers to PMTCT programmes in sub-Saharan Africa. Packages of solutions to address barriers at different levels are likely to be the most effective [33]

**Laboratory tests:** All HIV-infected pregnant women attending the Muhima health centre were routinely offered the following screening tests: serum for ABO/Rh (D) type, hemoglobin, rapid plasma reagin (RPR), CD4 count and urine dipstick for glucose and protein. All these tests were performed by the Muhima hospital laboratory that was equipped with CD4 machine and run by laboratory technicians, under the immediate supervision and mentorship of the National Reference Laboratory team, also responsible for quality control [16]. From the health system perspectives, there was no major workload-specific bottleneck during the study period (36 months), as the laboratory would receive requests for CD4 cell count tests for about 20 HIV-infected pregnant women per month on average (700 women in total) [34, 35].

**Financial access to MPTCT services:** MCH/PMTCT services as provided at health centre, district and tertiary levels (e.g. antenatal care, HIV testing & counselling, ARV drugs, childbirth/newborn & postnatal care, immunization, laboratory tests, family planning) were all integrated into the broader community-based health insurance framework. Clients with valid membership were granted free access (only 10% co-payment required) to the full range of needed PMTCT - related interventions at any point of service. Since 2005, with the support of development partners and particularly the Global Fund to fight AIDS, Malaria & Tuberculosis, the Government of Rwanda has been implementing a specific funding mechanism, with the goal of ensuring subsidized membership fees (USD two dollars/person/year) for the community-based health insurance benefits to the most vulnerable families, including people living with HIV, widows and orphans. These were identified at village level through community participation process, under the leadership of local authorities. This approach was part of the Rwanda government efforts to provide adequate response to the HIV/AIDS pandemic - a national health policy towards universal access to HIV prevention, treatment and care that has been in place for the last decade, with a special focus on equity, as emphasized by the Global Plan [3, 36–38].

**Data management:** During the study period (2007-2010), each woman, with confirmed HIV infection, was allocated one single identification number (ID), as part of the TRACnet database - an electronic health record system, launched in 2005 by the Treatment and Research AIDS Center (TRAC/Rwanda Ministry of Health) [25]. The unique patient ID number was a key element for linking various PMTCT interventions provided both to the mother and the infant during the follow up period. Other PMTCT-related socio-demographic, clinical and biological data were recorded manually in logbooks used for the following units: antenatal care, childbirth/operative delivery (mode of delivery), postnatal care and laboratory. These separate registers used in Muhima did not provide appropriate platform for optimal data synthesis and analysis to identify gaps in programme performance. To address this specific need, WHO promotes the use of integrated monitoring tool such as the “Three interlinked Patient Monitoring Systems” that is meant for supporting integrated service provision, follow up of mother-infant pair, monitoring of core maternal and infant indicators and key HIV/TB & Malaria-related variables [39, 40].

A key contribution of this study has been to describe specific operational approaches that were applied at primary health care setting level to support implementation and scale-up of PMTCT interventions. Although its findings corroborate those from several studies in similar settings, including the incidence rate of HIV-1 mother - to - child transmission at 6 weeks of life, it was only conducted in one of the 30 Rwandan districts and located in urban area. The results would not be generalizable to the entire country.

## Conclusion

The Muhima cohort study provided a great opportunity for an active monitoring process to ascertain outcomes, including the incidence of HIV-1 MTCT at 6 weeks of age, reflecting in utero or intrapartum transmission, among 679 babies born to HIV-1 infected mothers and followed up at primary health care level. Of the 700 mother - infant pairs registered in the study, the observed transmission rate was 3.2%, with a minimal proportion of eligible participants (3%) lost to follow - up. Disclosure of HIV status to partner was the major factor significantly associated with HIV-1 status of infants at 6 weeks of age. The Muhima type of decentralized health facility offered an appropriate platform and feasible strategy for scaling up effective PMTCT interventions, with the following key national policy considerations: family - centred approach; integrated service delivery for PMTCT/MCH interventions, including community-based approach; task shifting; subsidized membership fees for people living with HIV, allowing for access to the full package of PMTCT/MCH services, under the community-based health insurance coverage. However, further research is needed to identify most cost - effective data management systems to support optimal integration, utilization and quality of PMTCT services for mothers and their babies, in the context of elimination of MTCT.
